# Aqueous Extract of *Adansonia digitata* reversed Cotton Seed Extract-Induced Testicular Damage in Wistar rats

**DOI:** 10.5935/1518-0557.20200092

**Published:** 2021

**Authors:** Dare Joseph Babatunde, Olaniyan Olugbemi Tope, Oigiagbe Eboselume Fidelis, Okotie Gloria Enevwo, Eweoya Olugbenga

**Affiliations:** 1 Department of Anatomy, Osun State University, Osogbo, Nigeria; 2 Laboratory for Reproductive Biology and Developmental Programming, Department of Physiology, Edo University Iyamho, Edo State, Nigeria; 3 Department of Anatomy, Bingham University Karu, Nasarawa State, Nigeria; 4 Department of Physiology, University of Ibadan, Ibadan, Nigeria; 5 Department of Anatomy, School of Medicine and Allied Health Sciences, University of the Gambia, Serrekunda, The Gambia

**Keywords:** Gossypol, Adansonia. Digitata, Biochemical assay, Testis, Wistar rats

## Abstract

**Objective:**

Infertility is the inability of sexually active couples without using birth control to get pregnant after one year of uninterrupted sexual intercourse. Cotton Seed Extract (CSE) has been linked to male infertility by causing oxidative damage to the testes due to the action of its active component, Gossypol. *Adansonia digitata* has been known to have many medically useful properties, including antioxidant effects. This study aimed at evaluating the effects of Adansonia digitata on Cottonseed extract-induced testicular damage.

**Methods:**

Forty (40) Adult male Wistar rats were divided into 8 groups of five rats per group (n=5). Group 1 served as the control and received 0.5 ml of phosphate buffer orally; Group 2 received 800 mg/kg b.wt A. digitata orally; Group 3 received 300 mg/kg b.wt Vitamin E only orally; Group 4 received 60 mg/kg b.wt CSE intraperitoneally; Group 5 received 20 mg/kg b.wt CSE intraperitoneally; Group 6 received 60 mg/kg b.wt CSE intraperitoneally and 800 mg/kg b.wt A. digitata orally; Group 7 received 20 mg/kg b.wt CSE intraperitoneally and 800 mg/kg b.wt A. digitata orally; Group 8 received 60 mg/kg b.wt CSE intraperitoneally and 300 mg/kg Vit. E orally. It was administered for 21 days. The testes and epididymis were dissected following abdominal incision. The epididymis was used for semen analysis while the testes was processed for histological analysis and biochemical assay. All the data was analyzed by ANOVA, using the SPSS version 17.0 software. A *p*<0.05 was considered significant.

**Results:**

CSE administration caused significant (*p*<0.05) decrease in sperm count, found in the group treated with CSE only. However, the Administration of *A. digitata* caused significant increase (*p*<0.05) in sperm count, G6PDH, LDH, GPx and SOD; however, MDA levels were decreased. Histological observations showed a decrease in the number of Spermatogonia and differentiating cells in the testes of rats treated with CSE.

**Conclusions:**

The results obtained revealed the antioxidant ability of *A. digitata* in counter-acting the testicular damage caused by CSE administration.

## INTRODUCTION

Infertility is the inability of a sexually active couple not using birth control to get pregnant after one year of uninterrupted sexual intercourse. It has been reported that male problems may play a role in 30 to 50 % of infertile couples (WHO, 2010). Cottonseed extract contains a yellow polyphenolic binaphthalene pigment, gossypol (Coutinho, 2002). Gossypol [(2, 2’-binaphthalene)-8, 8’-dicarboxaldehyde-1, 1’, 6, 6’, 7, 7’-hexahydroxy-5,5’-diisopropyl-3,3’-dimethyl] is a naturally occurring compound extracted from the cotton plant and has been extensively studied as an oral male contraceptive agent (Shelley *et al.,* 2009). Gossypol has been referred to as a male contraceptive; however, it does not affect sex hormone levels or libido, and its mechanism is distinct from that of steroidal oral contraceptives used by women (Oyetunji *et al.,* 2012). Gossypol exerts its contraceptive action by inhibiting an enzyme that plays a crucial role in energy metabolism in the sperm and spermatogenic cells. The target enzyme, lactate dehydrogenase X (LDH-X), is found only in sperm and in male gonadal cells involved in glycolysis, and it plays a role in inducing mitochondria to produce energy (Oyetunji *et al.,* 2012). Gossypol has been shown to cause testicular damage via inhibition of acrosomal enzymes (Yuan & Shi, 2000), affinity for extracellular and intracellular proteins (Wang *et al.,* 1992), and inhibition of spermatogenesis and sperm motility (Countinho, 2002). The mitochondria of the target germ cells is the most sensitive and the most severely damaged among cellular organelles in response to gossypol (Xue *et al.,* 1983). The damages include the swelling, vacuolation, crista depletion, lysis, granular accumulation in matrix and the process of intact mitochondria disintegration (Xue *et al.,* 1983). The activity of the mitochondrial marker enzyme, the LDH-X of human spermatozoa, was markedly decreased or suppressed completely after gossypol treatment (Xue *et al.,* 1983).

Plants have been used to treat oxidative damage in recent years. A variety of antioxidant plants have been assessed for their ability to counteract oxidative stress created by alternative mechanisms in the testes, for example lycopene, the red plant antioxidant that is a major constituent of tomatoes, is capable of reversing the oxidative damage induced in rat testes following exposure to cyclosporin A or cisplatin (Aitken & Shaun, 2009). Other plants have the *lycium barbarum* herb, which defends the testes from oxidative damage induced by heat stress and hydrogen peroxide; and *Musa Paradisiaca*, which protects the testes from oxidative testicular damage resulting from induced diabetes (Aitken & Shaun, 2009). Baobab or *Adansonia digitata* can be used as one of such plants due to its antioxidant properties. *Adansonia digitata* belongs to the Malvaceae family (Bremer *et al.,* 2003). Its distribution area is large, and this species can be found in most of Sub-Sahara Africa’s semi-arid and sub-humid regions as well as in western Madagascar (Diop *et al.,* 2006). Baobab is a very long-living tree with multipurpose uses. Its different plant parts are widely used as food and medicine (Sidibe & Williams, 2002). Its fruit pulp has very high vitamin C content, and can be used in seasoning, as an appetizer and to make juices, its seeds contain appreciable quantities of crude protein, digestible carbohydrates and oil; whereas they have high levels of lysine, thiamine, calcium and iron (Caluwé *et al.,* 2010). The dry baobab fruit pulp has a slightly amount of tart, refreshing taste and is very nutritious, with particularly high values for carbohydrates, energy, calcium, very high potassium, thiamine and vitamin C (Arnold *et al.,* 1985). The high calcium contents of the fruit pulp make baobab fruits attractive as a natural source of calcium supplementation for pregnant and lactating women, as well as for children and the elderly (Osman, 2004). Because of its high natural vitamin C content, baobab fruit pulp has a well-documented antioxidant capability (Brady, 2011). Antioxidants could help prevent oxidative stress related diseases such as cancer, aging, inflammation (Ramadan *et al.,* 1994) and cardiometabolic diseases, since they may eliminate free radicals, which contribute to these chronic diseases (Carlsen *et al.,* 2010). These activities may be attributed to the presence of sterols, saponins, triterpenoids, flavonoids, phenolic compounds (Chadare *et al.,* 2009) and triterpenes in the aqueous extract (Brady, 2011).

This study is aimed at evaluating the effects of *Adansonia digitata* on Gossypol- induced testicular damage in Wistar rats.

## MATERIALS AND METHODS

Forty (40) male Wistar rats weighing between (150 - 200g) were obtained from the animal housing facility of Bingham University Karu, divided into eight groups (n=5). The rats were in the control room and acclimatized for two weeks before the experiment commenced. They were provided with rat pellets (growers mash) and water *ad libitum*. The ethical approval on animal act right was obtained from the Institutional Animal Care Committee of the same Institution. All the experimental procedures were done following the experimental guidelines of the Institution’s Animal Ethics Committee (IAEC) of Bingham University, Karu, Nasarawa State, Nigeria.

### Experimental Animals and Handling

The rats were grouped into eight (8) groups of 5 rats each;

Group 1 received 0.5 ml of phosphate buffer saline (PBS)

Group 2 (800 mg/kg b.wt *Adansonia* only)

Group 3 (300 mg/kg b.wt Vitamin E), 

Group 4 (60 mg/kg cottonseed extract only),

Group 5 (20 mg/kg cottonseed extract only),

Group 6 (60 mg/kg cottonseed extract + 800 mg/kg b.wt *Adansonia*),

Group 7 (20 mg/kg cottonseed extract + 800 mg/kg b.wt *Adansonia*)

Group 8 (60 mg/kg cottonseed extract + 300 mg/kg b.wt Vitamin E).

### Experimental protocol

#### • Extracts Preparation

Cottonseed was obtained from the Nyanya market and authenticated in the Biological Science laboratory, Bingham University. The residual cotton fibers were picked off and the seeds were grinded. 20 g of the powdered cottonseed were soaked in 200 ml of 70% ethanol. The solution was left to settle for 24 hours with occasional stirring using a glass rod. The solution was filtered after 24 hours using filter paper and the residue was discarded according to Ramadan *et al.* (1994). The filtrate obtained was concentrated using a hot plate at 60^0^C until a yellowish semi solid paste was obtained, which was dissolved in phosphate buffer for intraperitoneal administration.

*The Adansonia* fruit pod was obtained from from the Nyanya market in Nassarawa state and authenticated in the Biological Science Laboratory, Bingham University. The fruit pod was broken to expose the pulp and seeds. 500g of the pulp was soaked in 5 liters of distilled water for 48 hours. The solution was then filtered using filter paper and the residue was discarded. The filtrate was concentrated at 60ºC, until a dark colored semi solid paste with a fruity smell was obtained. This was dissolved in phosphate buffer for oral administration.

### • Extracts Administration

*Adansonia digitata* was administered by oral gavage. Proper concentrations of *Adansonia digitata* and vitamin E were administered through a metal oropharyngeal cannula. The LD50 of *Adansonia digitata* is 8000 mg/kg according to Ramadan *et al.* (1994). The administration of cottonseed extract was via intraperitoneal injection for a period of 21 days.

### • Sample Collection

The animals were slaughtered by cervical dislocation 24 hours after a period of 21 days of the last administration, and their testes were excised. The left testes from each rat was fixed in Bouins fluid for histological analysis, using H&E stains while the other was homogenized in 5% sucrose solution for enzyme histochemistry. The spermatozoa were obtained from the caudal part of the epididymis for semen analysis using the new improved nebular haemocytometer counting chamber.

### Biochemical Procedures

#### • Semen Analysis

The caudal epididymis was dissected out; several incisions (1mm) were made in the caudal epididymis, which was suspended in 1ml of normal saline solution according to Olaniyan *et al*. (2018) for sperm motility and morphology. The sperm concentrations were determined by fixing the sperm in 10% formo-saline in a 1:9 ratio. The counting was done using the newly improved nebular hemocytometer (Olaniyan *et al*., 2018).

### • Histological preparation

The histology of the testes was carried out by modifying the method described by Akpantah *et al.* (2003). The organs were cut in slabs about 0.5 cm thick transversely and fixed in bouins fluid for one day, after which it was transferred to 70% alcohol for dehydration. The tissues were passed through 90% and absolute alcohol, and xylene for different durations before they were transferred into two changes of molten paraffin wax for 1 hour each in an oven at 65^0^C for infiltration. They were subsequently embedded and serial sections were cut using rotary microtome at five microns (5 µm). The tissues were transferred onto albumenized slides and allowed to dry on a hot plate for 2 minutes. The slides were then de-waxed with xylene and passed through absolute alcohol (2 changes); 70% alcohol, 50% alcohol and then water for 5 minutes. The slides were then stained with Hematoxylin and Eosin. The slides were mounted in Canada balsam. Photomicrographs were taken using X100 and X40 magnifications for each group.

### • Enzyme Histochemistry

The excised testicular tissues were put in Lao style mortar containing 1 ml of 0.25 mm (5%) sucrose solution and were homogenized thoroughly. Tissue homogenates were collected in 5ml plain serum bottle for enzyme assay; Glucose 6 phosphate dehydrogenase (G6PD), Superoxide Dismutase (SOD), Malondialdehyde (MDA), Glutathione Peroxidase (GPx) and Lactate Dehydrogenase (LDH).

### • Malondialdehyde (MDA) level determination in tissue homogenates

Malondialdehyde levels in tissues were measured according to the protocol outlined by Stocks & Domandy (1971), as shown below. 0.1 ml of homogenate was pipetted into a plastic test tube. 1 ml of 20% trichloroacetic was added to it. The mixture was mixed and centrifuged at 2000g for 5 minutes. 0.5 ml of the supernatant was pipetted into a Pyrex test tube. 0.05 ml of 10.0 µmol/L of 1, 1, 3, 3-Tetramethoxylpropane was pipetted into another Pyrex test tube (standard). 0.5 ml of Trichloroacetic acid solution and 1.0 ml of Thiobarbituric acid were pipetted into a 3rd Pyrex test tube (blank). All tubes were stoppered tightly. The test tubes were heated in a water bath at 100^0^C for 20 minutes. All tubes were cooled in water. The spectrophotometer was blanked using the reagent blank at 532 nm. Tests and standards absorbance were read.

$$\mathrm{Tissue}\;\mathrm{Malondialdehyde}=\frac{\left(\mathrm{Absolute}\;\mathrm{of}\;\mathrm{test}\times 10\right)}{\mathrm{Absolute}\;\mathrm{of}\;s\tan\mathrm{dard}}\times 10µ\mathrm{mol}/L$$

### • Determination of Glucose 6 Phosphate Dehydrogenase (G-6-PDH) Activity in tissue homogenate

We measured the G6PD activity in the homogenate using the method of Lohr & Walker (1974). The procedure is as shown in the reactive table (table 1).

**Table 1:** Reactive table

Reagent R2 (buffer)	1.0 ml
Reagent R2 (NAOP)	30 µl
Homogenate	15 µl
Mix, incubate for 5mins at 370C	
R3 (Glucose 6 phosphate)	15 µl

Read initial absorbance at 365 nm and start timer simultaneously (against air), read the absorbance again after 1, 2 and 3 minutes.

### • Calculation

G6PDH in homogenate activity = 60571xrate of change in absorbance per min (mµ/ml).

G6PDH in U/L homogenate = 60571xrate of change in absorbance per min x1000.

### • Determination of Lactate Dehydrogenase (LDH) Activity in Tissue Homogenate

We measured the LDH activity in the homogenate according to the method from Balch *et al*. (2009). The homogenate was centrifuged at 10000xg for 10 minutes at 4ºC. The clear supernatant obtained was used for the measurement of LDH activity.

### • Determination of Glutathione Peroxidase activity in tissue homogenate

Glutathione peroxidase activity in homogenate was determined using the method of Paglia & Valentine (1967), with the Randox lab, Ardmore, Diamond road, Crumlin co, UK BT294QY reagent kit.

### • Determination of Superoxide Dismutase (SOD) Activity in the homogenate

The enzyme activity of SOD was assayed according to the method of Misra & Fridovich (1972), using the reagent kit produced by Randox lab Ltd.

### Statistical Analysis

We statistically evaluated the data using the One-way ANOVA (Analysis of Variance) with the SPSS/17.0 software (SPSS Inc, Chicago, USA). The Tukey post-hoc analysis was expressed as Mean ± Standard deviation. A *p* value <0.05 was considered statistically significant.

## RESULTS

### Semen parameters

The results obtained indicate that there is no significant difference (*p*>0.05) in sperm count and motility between the control, *Adansonia digitata* and Vitamin E groups. Administration of Adansonia Digitata and Vitamin E, as shown in table 2, maintained the Spermatogenic characteristics as reflected in the sperm concentration and in the viability or motility compared with cottonseed extract treated groups. Significant reduction in sperm counts and motility characterized the sperm quality in the cottonseed groups, particularly at higher 60 mg/kg body weight of CSE. The results showed greater impact on the sperm motility among the animals that received CSE, with significant decrease (*p*<0.05) in sperm count and motility when compared to the control group, and 20 mg/kg body weight of CSE. The group treated with both Cottonseed extract and *Adansonia digitata* showed a significant improvement (*p*<0.05) in sperm count and motility compared with Cottonseed extract treated groups (table 2). The intervention of Adansonia Digitata and Vitamin E in groups 6, 7 and 8 revealed sperm characteristics similar to both the positive and negative control groups. Significant increase in sperm concentration (counts) and motility defines the animals groups compared with the groups that received only CSE.

**Table 2:** Effects of Aqueous Extract of Adansonia digitata on sperm parameters in cottonseed extract-induced testicular damage in Wistar rats

Group	Sperm count (×106/ml)	Motility (%)
1 (PBS Only)	84.00±3.50	70.00±1.12
2 (Ad only)	82.00±11.70	60.00±0.98
3 (Vit. E only)	89.00±4.50	65.00±2.31
4 (60mg/kg CSE)	63.00±4.20^+^	5.00±1.94^+^
5 (20mg/kg CSE)	69.00±1.00^+^	10.00±2.01^+^
6 (60mg/kg CSE +Ad)	78.00±22.60*	60.00±1.11*
7 (20mg/kg CSE +Ad)	81.50±0.70*	50.00±1.06*
8 (60mg/kg CSE +Vit. E)	96.00±2.80*	60.00±0.04*

Values are represented in mean±SEM, n=5

* shows *p*<0.05 level of significance when compared with CSE groups + shows *p*<0.05 level of significance when compared with control group.

The results obtained indicate that there is no significant difference (*p*>0.05) in G6PDH activity between the control, *Adansonia digitata* and Vitamin E groups. However, the cottonseed extract-treated groups revealed a significant difference (*p*<0.05) in the activities of G6PDH enzymes when compared to the control group. The LDH levels were also significantly decreased (*p*<0.05) in the group treated with cottonseed extract compared to the control group. The group treated with cottonseed extract and *Adansonia* extract showed a significant increase (*p*<0.05) in LDH activity compared with the cottonseed extract groups (table 3). These observations showed significant decreases in the G-6-PDH and LDH enzyme activities in 60 mg/kg body weight of the animal in CSE compared with the 20 mg/kg body weight of CSE and the negative controls. A more important point of interest is the significant increase in G-6-PDH and LDH enzyme activities in the groups with Adansonia Digitata and Vitamin E that were presented with similar characteristics with the control groups.

**Table 3:** Effects of *Adansonia digitata* Aqueous Extract on the Activity of Carbohydrate Metabolic Enzymes in Gossypol-Induced Testicular Damage in Wistar rats

Group	G6PDH (IU/L)	LDH (IU/L)
1 (PBS Only)	5625± 2.90	4030±3.80
2 (Ad only)	5378± 5.70	4121±4.30
3 (Vit. E only)	5331± 3.90	4027±1.50
4 (60mg/kg CSE)	3150± 3.40^+^	2941±2.70^+^
5 (20mg/kg CSE)	2753± 2.90^+^	2769±4.40^+^
6 (60mg/kg CSE +Ad)	4784± 7.50*	3964±3.50*
7 (20mg/kg CSE +Ad)	4645± 2.40*	3041±1.80*
8 (60mg/kg CSE +Vit. E)	4525± 3.20*	3647±5.20*

Values are represented in mean±SEM, n=5 G6PDH = Glucose 6 phosphate dehydrogenase, LDH = Lactate dehydrogenase

* shows *p*<0.05 level of significance when compared with CSE groups + shows *p*<0.05 level of significance when compared with control group

### Lipid Peroxidation (Malondialdehyde levels)

From the results on table 4, there is a significant increase (*p*<0.05) in malondialdehyde levels in the cottonseed extract treated groups when compared to controls. The group treated with both cottonseed extract and *Adansonia digitata* showed a significant decrease (*p*<0.05) in malondialdehyde levels compared with the cottonseed extract group. Malondialdehyde (MDA) activities had been subjected to lipid peroxidation; therefore, increased MDA activities in the CSE treated animals means increased lipid peroxidation with significant presentation in the 60mg/kg CSE compared to 20mg/kg CSE.

**Table 4:** Effects of Aqueous Extract of *Adansonia digitata* on lipid peroxidation, Superoxide Dismutase (SOD) and Glutathione Peroxidase (GPx) activity in Gossypol-Induced testicular damage in Wistar rats.

Group	MDA (µmol/L)	SOD(U/ml)	GPX (nmol/min/ml)
1 (PBS Only)	21.00±0.60	884±3.00	704±2.90
2 (Ad only)	23.70±0.90	657±1.50	716±3.20
3 (Vit. E only)	23.30±1.50	764±3.40	651±2.20
4 (60mg/kg CSE)	29.70±1.20+	603±3.20+	581±6.10+
5 (20mg/kg CSE)	25.30±1.90+	616±4.40+	660±2.50+
6 (60mg/kg CSE +Ad)	17.00±1.20*	708±2.70*	764±2.00*
7 (20mg/kg CSE +Ad)	23.70±0.90*	681±2.10*	688±3.00*
8 (60mg/kg CSE +Vit. E)	21.30±0.90*	626±3.80*	707±4.10*

Values are represented in mean±SEM, n=5 SOD = Superoxide dismutase, GPX = Glutathione Peroxidase

* shows *p*<0.05 level of significance when compared with CSE groups ^+^shows *p*<0.05 level of significance when compared with control group

### Antioxidant enzymes (SOD and GP_X_)

The groups treated with cottonseed extract only showed a significant reduction (*p*<0.05) in antioxidant enzymes (SOD and GP_X_) compared with the control group. The group treated with both cottonseed extract and *Adansonia digitata* revealed significant increase (*p*<0.05) in SOD and GPx, when compared with the cottonseed extract-treated groups (table 4). SOD and GPx have been shown to possess free radical mopping activities, CSE had proven its cell damage impact with the significant lowering in the SOD and GPx activities and, therefore, subjecting the tissue to oxidant effects, especially in 60mg/kg CSE compared to 20mg/kg CSE. The preventive antioxidant promoting effects of Adansonia Digitata and Vitamin E is reflected in the impact to maintain these antioxidant enzyme activities as shown on table 4

### Histological analysis

The H&E staining of the testicular tissue revealed that the group that received Adansonia Digitata showed normal cell configuration, but in the cottonseed treated group, there were abnormalities in testis integrity. The group that received both cottonseed extract with A. digitata demonstrated a significant improvement in testis cytoarchitecture. The administration of Adansonia Digitata maintained the histological appearances of the testes; the spermatogonia population well expressed all stages of the spermatogonial differentiation seen. The basement membrane, with the lining seminiferous epithelium are shown, as well as the three (3) basic types of cells expected in the seminiferous tubules (Spermatogonia, Sertoli and Leydig) clearly indicated as shown in plate B. Improve and continuous evidence of spermatogenesis; the interstitial cells of Leydig, located in compact interstitial spaces, maintenance of close seminiferous tubules apposition with the presence of Sertoli cells and the spermatogonia A and B is clear evidence of testicular integrity maintained by the administration of Adansonia digitata and Vitamin E, in relation to the control animals as shown in plates A, B and C. However, treatment with cotton seed ethanol extract, with the gossypol as the active component altered the testicular architecture, caused abnormal widening of the interstitial spaces with loss of the interstitial cells, especially for 60mg/kg CSE compared to 20mg/kg CSE, as per indicated in plates D and E. Reduction in the spermatogonia population and increase in the empty filled spaces an evidence of vacuolation is associated with the cottonseed extract treatment in both 20 and 60 mg/kg doses. However, intense degeneration was significant in the spermatogonia population as shown in plate D. More importantly, the loss of seminiferous epithelium because of alterations in the basement membrane degeneration could justify the empty lumen indicated in the cottonseed extract treated animals. Intervention of testicular damage by co-administration of cottonseed extract and Adansonia digitata revealed maintenance of testicular integrity and continuous evidence of spermatogenesis, as all the three major cells of seminiferous tubules clearly demonstrated and all stages of spermatogonia cell shown; the differentiating spermatogonia, Sertoli and the interstitial cell in the interstitial spaces. Vitamin E is a proven antioxidant that showed improved testicular architecture, similar to results from Adansonia Digitata treatment, as depicted in plates F, G and H.

## DISCUSSION

The findings in this study showed that cottonseed extract exert a significant reduction in sperm parameters when compared to the control group. These observations support the report made by Randel *et al.* (1992) who revealed that polyphenolic binaphthalene pigment (gossypol) in cottonseed extract act as a natural contraceptive in reducing the energy level and mitochondrial biomarker in sperm cell. The group treated with both cottonseed extract and *Adansonia digitata* showed a significant increase in sperm count and motility, compared with cottonseed extract-treated groups (table 2). This may be attributed to the active constituents of the extract such as triterpenoids, flavonoids, and vitamin C, enhancing the energy level and mitochondrial biomarker in the sperm.

The results obtained from Table 3 indicate that there was no significant difference between the G6PDH activity of the controls, the *Adansonia digitata* and Vitamin E-treated groups. However, the administration of cottonseed extract caused a significant dose-dependent reduction in G6PDH activity, suggesting that the cottonseed extract suppresses the antioxidant system in the testis. This result is in line with that obtained by Bender *et al.,* (1988) who revealed that gossypol could generate free radicals from testicular tissue and reduce its antioxidant defense. The groups treated with cottonseed extract and *Adansonia digitata* showed a significant increase in G6PDH activity when compared with cottonseed extract only. This suggests that *Adansonia digitata* (baobab) boosted the G6PDH antioxidant system of the testis, which is in agreement with the result obtained by Carlsen *et al.* (2010), who showed that the active components of *Adansonia digitata* such as triterpenes, phenolic, flavonoids and saponins might be responsible for its biological activity. G6PDH is a cytosolic enzyme in the pentose phosphate pathway that supplies reducing energy to cells, by maintaining the level of the NADPH, thereby enhancing glutathione functions in the cells. The G6PDH enzyme function in to catalyze the oxidation of glucose-6-phosphate to 6-phosphogluconate, while concomitantly reduce nicotinamide adenine dinucleotide phosphate NADP+ to NADPH; or, in terms of electron transfer, glucose-6-phosphate loses two electrons to become 6-phosphogluconate, and NADP+ gains two electrons to become NADPH, which is the first step in the pentose phosphate pathway. In addition to producing the 5-carbon sugar ribose, G6PD is also responsible for maintaining adequate levels of NADPH inside the cell, which is required as cofactor for many biochemical reactions, and for keeping glutathione, a tri-peptide, in its reduced form (Jacobasch *et al.,* 1982).

**Figure:**
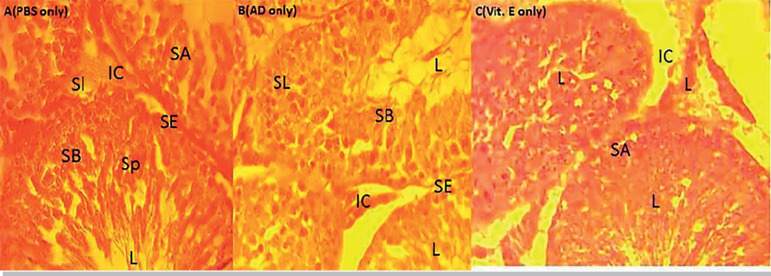
**Plate A.** Demonstrates testis of animal treated with o.5ml PBS only (control); all the stages of the spermatogonia population expressed, SB-Spermatogonia, SA- Spermatogonia B, SE-Seminiferous Epithelium, IC-Interstitial Cells, SL- Sertoli Cells. H&E stain, x400 **Plate B.** Indicates testis of animal treated with 800mg/kg A.D.; the seminiferous tubules closely apposed and all the stages of the spermatogonia population expressed, SB- Spermatogonia, SA-Spermatogonia B, SE-Seminiferous Epithelium, IC-Interstitial cells, SL- Sertoli cells. Indication of continuous spermatogenesis with the presences of spermatocytes in the lumen H&E stain, x 400 **Plate C.** Indicates testis of animal treated with 300mg/kg Vit. E; Loosening of the seminiferous tubules close apposition, mild alteration in the all architecture seen in the loosening of the SE-Seminiferous Epithelium. However, all the stages of the spermatogonia population expressed, SB-Spermatogonia B, SESeminiferous Epithelium, IC- Interstitial cell, SL- Sertoli cells. Indication of continuous spermatogenesis with the presence of spermatocytes in the lumen H&E stain, x 400

**Figure:**
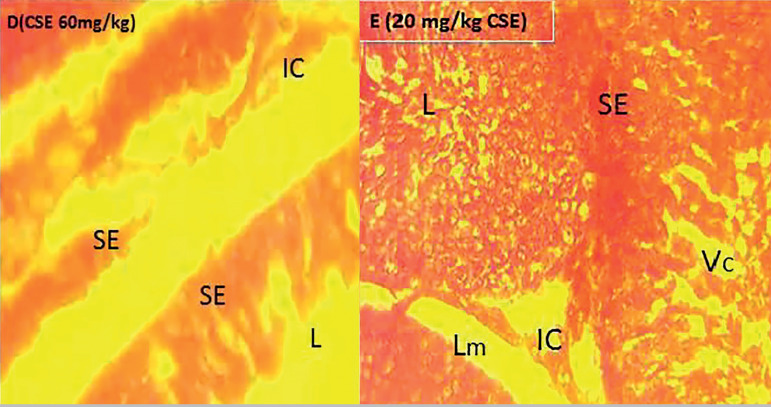
**Plate D.** Demonstrates testis of animals treated with 60mg/kg CSE; Severe loss of spermatogonia population, abnormal widening of the lumen and the interstitial spaces due to loss of interstitial cells, damage to the basal membrane and loss of SE-Seminiferous epithelium are evident H&E stain, x400 **Plate E.** Demonstrates testis of animal treated with 20mg/kg CSE only, increase in interstitial spaces with loss of cells, loose basal membrane (Lm) and spermatogonia degeneration with vacuolation. H&F stain, x 400

**Figure:**
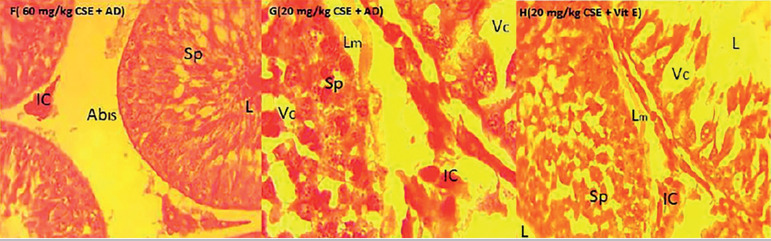
**Plate F.** Demonstrates testis of animal treated with 60mg/kg CSE+AD; abnormal widening of Interstitial spaces, Abis, however, all the stages of spermatogonia population expressed, SB-Spermatogonial, SASpermatogonia B, SE-Seminiferous Epithelium, IC-interstitial cells, SL-Sertoli cells. Indication of continuous spermatogenesis with the presences of spermatocytes in the lumen H&E, x400 **Plate G.** Demonstrates testis of animal treated with 20mg/kg CSE+AD; Reduced abnormal widening of interstitial paces, mild alteration in the all architecture seen in the loosening of the SE-Seminiferous Epithelium, reduced evidence of vacuolation; however, with spermatogonia regeneration and continuous spermatogenesis. H&E stain, X1000. **Plate H.** Demonstrates testis of animals treated with 60mg/kg CSE+Vit. E; Spermatogonia population with evident of regeneration and a reduced vacuolation. Interstitial spaces intact with the Leydig cells and reduced loose basal seminiferous membrane with continuous spermatogenesis. H&Stain, x 400

Cottonseed extract also decreases LDH levels significantly when compared with the Adansonia-treated group. The group treated with cottonseed extract with *Adansonia digitata* had a significant increase in LDH activity, as compared with the cottonseed extract-treated group. This is in agreement with the study conducted by Ikeda (1990), who demonstrated that gossypol is able to reduce the level of LDH by inhibiting glycolysis and acrosomal enzymes level, causing overall reduction in mitochondrial marker. Malondialdehyde is often considered as the major hallmark of lipid peroxidation damage, which causes secondary damage to cell functions (Korchazhkina *et al.,* 2003). In Table 4, there was a significant increase in malondialdehyde levels in the groups treated with cottonseed extract when compared with the *Adansonia digitate-*treated group. This finding tallies with that from Zhang *et al.* (2011), who demonstrated that *Adansonia digitata* is rich in vitamin C, thiamine, lysine, phenolic compounds, triterpenoids, flavonoids, saponins and sterols, which are able to scavenge free radical in any biological environment. The group treated with cottonseed extract with *Adansonia digitata* showed significant decrease in malondialdehyde levels, as compared with the cottonseed extract-treated group.

Superoxide dismutase (SOD) is closely related to the cellular oxidative metabolism, which widely exists in vivo. It is a natural scavenger of reactive oxygen species (Zhang *et al.,* 2011). SOD specifically combines with superoxide anions and acts synergistically with glutathione peroxidase to prevent cell membrane lipid peroxidation and the formation of metabolites by directly capturing and eliminating free radicals (Alul *et al.,* 2003). Glutathione peroxidase (GPx) has a strong ability to scavenge lipid peroxide and hydrogen peroxide by converting hydrogen peroxide (H_2_O_2_) into H_2_O. From the results shown in Table 3, cottonseed extract showed a significant decrease in the antioxidant level, as compared with controls, while the group that received both cottonseed extract with A. digitata demonstrated a significant increase in antioxidant activity.

The testicular tissue revealed that the group that received Adansonia Digitata showed normal cell configuration, but in cottonseed-treated group, there was testis integrity abnormality. The group that received cottonseed extract with A. digitata demonstrated a significant improvement in testis cytoarchitecture. Dare *et al.* (2017) reported an abnormal widening of the interstitial space, loss of the basal lamina, spermatogonia degeneration with vacuolation. Loss of seminiferous tubules germinal epithelium were also seen in animals exposed to X-ray. They noted that exposure to X-ray disrupts spermatogenesis by disruption and depletion of spermatids and spermatogonia population, which caused increase in testicular tissue damage and, consequently, altered the sperm characteristics. These similar findings repeated in the histological characteristics of animals treated with the cottonseed extract. Therefore, as reported by Wang *et al.* (1992), Yuan & Shi (2000), and Coutinho, 2002 in their toxicology studies, Gossypol has been shown to cause testicular damage via inhibition of acrosomal enzymes, affinity for extracellular and intracellular proteins, inhibition of spermatogenesis and sperm motility.

The mitochondria of the target germ cells were the most sensitive and the most severely damaged among cellular organelles in response to gossypol (Xue *et al.,* 1983). The damages included the swelling, vacuolation, crista depletion, lysis, granular accumulation in the matrix and the process of intact mitochondria disintegration (Xue *et al.,* 1983). The activity of the mitochondrial marker enzyme, the LDH-X of human spermatozoa, was markedly decreased or suppressed completely after gossypol treatment (Xue *et al.,* 1983). Findings from the study show that the cottonseed extract administered at 60 mg/kg per body weight had more detrimental effects on the male reproductive functions as compared to 20 mg/kg.

## CONCLUSION

*Adansonia Digitata* seems to have fertility-boosting effects on cottonseed extract-induced testicular dysfunction. It is known that the *Gossypol* present in cottonseed extract has antifertility effects due to its role in exposing the testes to oxidative damage. Through this model, we have been able to demonstrate that the administration of *Adansonia Digitata* could improve male reproductive functions through its antioxidant properties. More studies should be carried out to isolate the main active component in Adansonia Digitata in drug formulations.
